# Enhancing Shiga toxin detection using surface plasmon resonance a study of antibody immobilization strategies

**DOI:** 10.1038/s41598-025-01686-9

**Published:** 2025-05-16

**Authors:** Zahra Karbalaee, Ali Hossein Rezayan, Ramezan Ali Taheri, Seyed Ali Mirhosseini, Mohammad Barshan-Tashnizi

**Affiliations:** 1 Department of Nanobiotechnology and Biomimetics, School of Bioengineering, College of Interdisciplinary Science and Technology, University of Tehran, Tehran, Iran; 2 Nanobiotechnology Research Center, New Health Technologies Institute, Baqiyatallah University of Medical Sciences, Tehran, Iran; 3 Applied Microbiology Research Center, Biomedicine Technologies Institute, Baqiyatallah University of Medical Sciences, Tehran, Iran

**Keywords:** SPR, *Shigella dysenteriae*, Stxb, Protein G, Shiga toxin, Oriented immobilization, Non-oriented immobilization, Biotechnology, Diseases, Nanoscience and technology, Optics and photonics

## Abstract

This study demonstrates enhanced detection of Shiga toxin (Stx), a key virulence factor in *Shigella dysenteriae*-induced bloody diarrhea, through optimized surface plasmon resonance (SPR) biosensor design. We present a comparative evaluation of antibody immobilization strategies, revealing significant advantages of protein G-mediated oriented immobilization over conventional covalent attachment. The covalent (non-oriented) approach using 11-mercaptoundecanoic acid-modified chip showed moderate performance (K_D_ = 37 nM, LOD = 28 ng/mL). In contrast, protein G-assisted orientation dramatically improved detection capabilities, achieving a 2.9-fold lower detection limit (9.8 ng/mL) and 2.3-fold higher binding affinity (K_D_ = 16 nM). Control measurements with free antibody-antigen interactions established a baseline affinity (K_D_ = 10 nM), demonstrating that the oriented method preserves 63% of native binding efficiency versus only 27% in the covalent approach. Mechanistic studies attribute these improvements to protein G’s ability to maintain optimal antibody orientation, thereby: (1) maximizing paratope accessibility, (2) minimizing steric interference, and (3) preserving binding site functionality. The 57% reduction in K_D_ relative to covalent immobilization confirms the method’s efficacy in maintaining antibody performance post-immobilization. These findings establish protein G-mediated orientation as the superior strategy for SPR-based Stx detection, offering substantial improvements in sensitivity and reliability for clinical diagnostics and food safety applications. The approach demonstrates particular promise for rapid, label-free detection of bacterial toxins in resource-limited settings.

## Introduction

Shiga toxin (Stx) and the closely related Shiga-like toxins are key virulence factors in diarrheal diseases and hemolytic-uremic syndrome (HUS). These toxins are primarily produced by *Shigella dysenteriae* and certain enterohemorrhagic *Escherichia coli* (EHEC) strains, which are Gram-negative pathogens of major public health concern^1,2^. Structurally, Shiga toxin is a homopentameric protein consisting of a single enzymatically active A subunit (Stxa) and a pentameric receptor-binding B subunit (Stxb). The B subunit facilitates toxin internalization by binding to globotriaosylceramide (GB3) receptors, which are widely expressed on host cells, thereby mediating the cytotoxic effects of Stxa^3^. Transmission occurs via contaminated food, water, or direct contact, underscoring the need for rapid and accurate diagnostic methods to enable timely intervention. Conventional techniques for detecting *Shigella*, EHEC, and their toxins include stool culture, polymerase chain reaction (PCR), immunoassays (e.g., ELISA, Western blot), and chromatographic or spectroscopic methods (e.g., HPLC, LC–MS)^2,4–7^. While reliable, these approaches are often labor-intensive, time-consuming, and costly, limiting their utility in resource-constrained settings. Furthermore, some methods lack the sensitivity for multiplex detection or real-time analysis, highlighting the demand for innovative alternatives. Biosensors have emerged as promising tools for toxin detection due to their specificity, speed, and cost-effectiveness. Various platforms, such as field-effect transistors (FETs)^8^, surface-enhanced Raman spectroscopy (SERS)^9^, and optical biosensors (e.g., SPR)^10^, have been explored, each with distinct advantages and limitations. FETs offer high sensitivity but may suffer from slow response times, while SERS requires complex sample preparation. In contrast, surface plasmon resonance (SPR) stands out as a label-free, real-time technique capable of monitoring biomolecular interactions with high sensitivity and minimal sample processing^11,12^. SPR-based biosensing has gained traction in clinical diagnostics and food safety due to its real-time monitoring, multiplexing capability, and quantitative precision^13,14^. Its applications extend to detecting bacterial toxins, pesticides, antibiotics, and other analytes, often surpassing traditional methods like ELISA in speed, sensitivity, and operational simplicity^15^. To enhance SPR performance—particularly for low-molecular-weight analytes (< 1 kDa)^16^—strategies such as fluorescence labeling^17^, gold nanoparticle (AuNP) amplification^18^, nanocomposite coatings^19^, and oriented antibody immobilization (e.g., via Protein G)^20^ have been developed. This study evaluates two antibody immobilization strategies—oriented (Protein G-mediated) vs. non-oriented—for SPR-based Shiga toxin detection. Protein G optimizes antibody positioning on the sensor surface, maximizing antigen-binding site availability. We assess the impact of this approach on sensitivity, limit of detection (LOD), and binding affinity (quantified by the dissociation constant, K_D_) under controlled laboratory conditions, aiming to establish both a robust platform for rapid, label-free toxin detection and a standardized framework for SPR-based Stx detection that can facilitate future applications in food safety testing and biosensor development.

## Methods

### Chemical and reagents

All chemicals were of analytical grade. 4-(2-Hydroxyethyl)−1-piperazineethanesulfonic acid (HEPES), sodium acetate, N-hydroxysuccinimide (NHS), N-(3-dimethylaminopropyl)-N'-ethylcarbodiimide hydrochloride (EDC), 11-mercaptoundecanoic acid (11-MUA), and Protein G were purchased from Sigma-Aldrich (St. Louis, MO, USA). Ethanolamine, ethylenediaminetetraacetic acid (EDTA), Tween 20, acetic acid, and sodium chloride (NaCl) were obtained from Fluka (Buchs, Switzerland). Deionized water (18.2 MΩ·cm resistivity) was used for all buffer preparations. For surface preparation, SPR gold disks were cleaned with piranha solution (3:1 v/v 98% H₂SO₄:30% H₂O₂; Caution: highly corrosive) followed by absolute ethanol. Chloroauric acid (HAuCl₄) and sodium citrate were sourced from Sigma-Aldrich. All glassware was sterilized with aqua regia (3:1 v/v HCl:HNO₃) and rinsed with deionized water. The following buffers were prepared for SPR experiments, Coupling buffer: 10 mM acetate buffer (pH 4.5; acetic acid/sodium acetate), Regeneration buffer: 15 mM NaOH with 0.2% (w/v) SDS and Running buffer: 10 mM HEPES, 150 mM NaCl, 3 mM EDTA, 0.005% (v/v) Tween 20 (pH 7.4). The Shiga toxin B subunit (Stxb; recombinant, non-toxic) and its corresponding mouse polyclonal antibody (anti-Stxb) were generously provided as a gift^21^. Structurally, Shiga toxin (Stx) comprises a catalytic A subunit (Stxa; 32 kDa) and a pentameric B subunit ring (Stxb; 5 × 7.7 kDa), where the B subunits mediate host cell binding via globotriaosylceramide (GB3) receptors.

### Instrumentation

The surface plasmon resonance (SPR) system employed in this study consisted of three core components: (1) a sensor chip, (2) a microfluidic delivery system, and (3) an optical detection unit. The sensor chip featured a layered structure with a 5 nm titanium adhesion layer and a 50 nm gold sensing surface (optimal thickness for plasmon excitation at 670 nm wavelength). Experiments were conducted using an AutoLab ESPRIT dual-channel SPR instrument (Metrohm Autolab, Utrecht, The Netherlands). The dual-channel configuration enabled simultaneous sample measurement and reference compensation, with the first channel serving as the active surface for antibody immobilization and subsequent analyte binding, while the second channel functioned as a reference to account for bulk refractive index variations, non-specific adsorption effects, and thermal drift (Fig. S1). This differential measurement approach effectively eliminated systemic artifacts from the binding signals. The system’s kinetic analysis module (Kinetic Evaluation Software v5.4, KE Instruments) processed all SPR data, which were recorded in real-time by the instrument’s data acquisition software (v4.3.1). Interaction profiles (Fig. S2) were generated through differential analysis between the two channels, eliminating systemic artifacts through the following equation:

$$\Delta {\text{RU }} = {\text{ RU}}_{{{\text{active}}}} - {\text{ RU}}_{{{\text{reference}}}}$$

where RU represents resonance units.

### Immobilization of Shiga toxin antibodies on an 11-MUA-modified chip

The immobilization procedure began with thorough cleaning of the sensor surface using piranha solution (3:1 v/v H_2_SO_4_:H_2_O_2_). The cleaned surface was subsequently immersed in 1 mM 11-mercaptoundecanoic acid (11-MUA) prepared in ethanol and left overnight at room temperature to allow formation of a self-assembled monolayer (SAM). Following SAM formation, the chip was rigorously washed three times with absolute ethanol followed by three washes with deionized water, then dried under nitrogen stream. The functionalized chip was carefully inserted into the SPR instrument. After completing optical alignment and verifying no leakage between SPR channels, the surface was stabilized by flowing acetate buffer (10 mM, pH 4.5) for 45 min. The carboxyl groups on the surface were then activated through treatment with a freshly prepared mixture of 400 mM EDC and 100 mM NHS for 300 s. Antibody immobilization was achieved by injecting anti-Stxb antibody solutions at varying concentrations (20, 40, and 100 μg/mL in acetate buffer) over the activated surface for 900 s to determine the optimal coating density. Any remaining active esters were subsequently blocked using 1 M ethanolamine (pH 8.5) for 600 s. Finally, the surface was treated with regeneration buffer (15 mM NaOH containing 0.2% SDS) for 120 s to remove any non-covalently bound material. Between each processing step, the surface was thoroughly rinsed with running buffer to remove excess reagents and ensure clean surface conditions for subsequent reactions. This comprehensive immobilization protocol enabled systematic evaluation of antibody surface density effects on the interaction signals.

### Oriented immobilization of Shiga toxin antibodies using protein G/antibody complex on an 11-MUA-modified chip

**Fig. 1:**
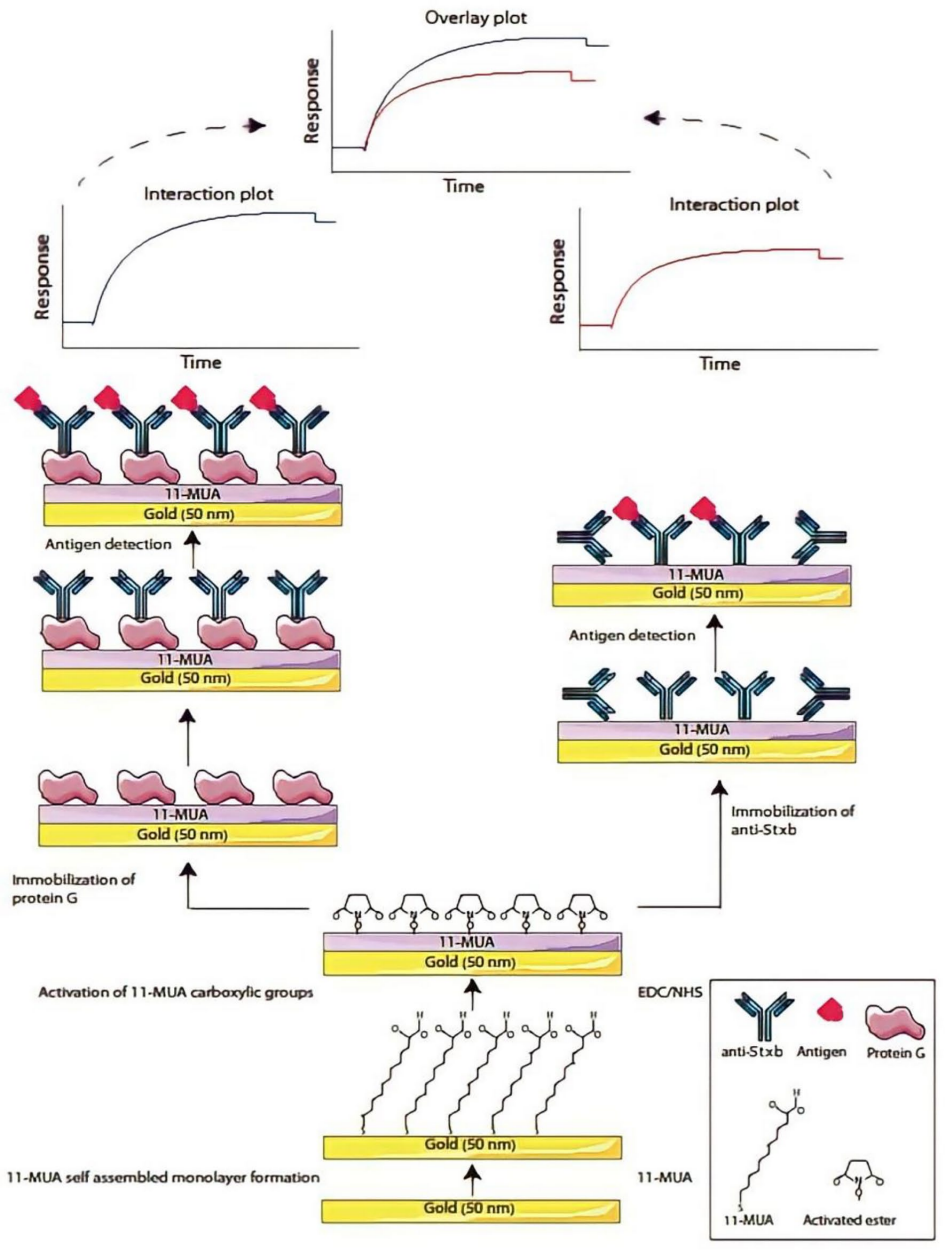
Schematic illustration of comparative study of direct detection of Shiga toxin with two immobilization methods. The interaction on the right is covalent immobilization of antibody on 11MUA modified sensor chip and the interaction on the left is oriented immobilization of antibody on 11MUA modified sensor chip by protein G.

The oriented antibody immobilization was achieved through a two-step procedure on an 11-MUA-functionalized sensor chip. Initially, the SPR gold chip surface was chemically cleaned with piranha solution (3:1 v/v H_2_SO_4_:H_2_O_2_) followed by thorough rinsing with deionized water. Subsequently, the surface was modified by overnight incubation in 1 mM 11-MUA ethanol solution to form a carboxyl-terminated SAM. Protein G (25 µg/mL) was first immobilized onto the 11-MUA-modified surface using standard amine coupling chemistry^22^, following the previously described immobilization protocol. This was followed by the introduction of anti-Stxb antibodies (40 µg/mL) as the secondary ligand, allowing the formation of oriented antibody/protein G complexes through specific Fc-region binding. The bioaffinity capture process ensured proper antibody orientation for optimal antigen binding. Figure 1 schematically compares this oriented immobilization approach with conventional non-oriented antibody attachment, highlighting the structural advantages of the Protein G-mediated method.

### Immobilization of Stxb antigen on an 11-MUA-modified chip

The Stxb antigen (40 µg/mL) was immobilized onto the 11-MUA-functionalized sensor chip following the identical protocol previously described for antibody immobilization. This enabled comparative evaluation of the two distinct anti-Stxb immobilization approaches (oriented vs. non-oriented) through quantitative analysis of their binding interactions with the surface-immobilized antigen. Key binding parameters, including the equilibrium K_D_, were calculated to assess and compare the binding affinities achieved by each immobilization strategy. The K_D_ values served as critical metrics for evaluating the functional consequences of antibody orientation on antigen capture efficiency.

### Biosensing protocol

For direct detection of Shiga toxin, anti-Stxb antibodies were first immobilized on the SPR gold-modified chip. Serial dilutions of the Stxb antigen (non-toxic Shiga toxin analog) were prepared in HEPES buffer and loaded into a 384-well microtiter plate. The detection protocol involved injecting 50 μL of each antigen concentration into both flow channels of the SPR sensor. Each binding cycle consisted of a 900-s association phase followed by a 600-s dissociation phase in running buffer. After each concentration measurement, the sensor surface was regenerated using a 10-min wash with 15 mM NaOH containing 0.2% SDS to completely remove bound analyte and prepare the surface for the next injection. The SPR sensor automatically recorded real-time binding responses, which were analyzed to determine key interaction parameters. K_D_ and maximum binding capacity (B_max_) were calculated from the binding curves. The LOD was determined using the formula LOD = 3.3σ/s, where σ represents the standard deviation of five replicate blank (HEPES buffer) measurements and s is the slope of the calibration curve^23^. This comprehensive approach enabled quantitative evaluation of the antibody-antigen interactions under controlled flow conditions.

## Results

### Immobilization of Shiga toxin antibodies on an 11-MUA-modified chip

**Fig. 2:**
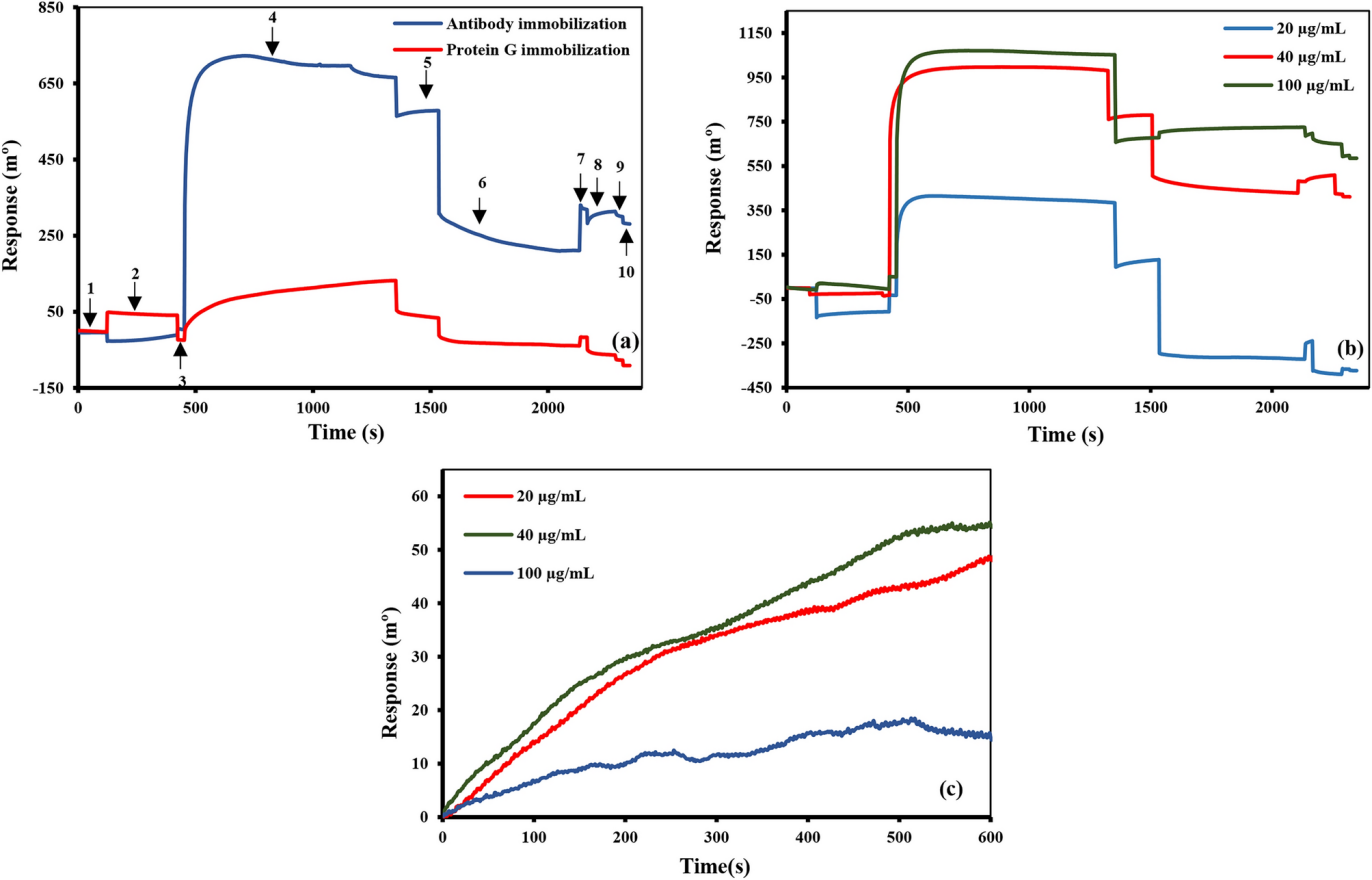
(**A**) Sensorgram showing the different stages of immobilization of 40 µg/mL anti-Stxb on 11-MUA SAM modified chip: 1) baseline, 2) surface activation with EDC/NHS, 4) washing, 4) antibody binding, 5) washing, 6) surface deactivation, 7) washing, 8) surface recovery, 9) washing, 10) return to the new baseline. (**B**) 10 step immobilization plot of different concentration of antibodies (100 µg/ml, 40 µg/ml and 20 µg/ml) on 11-MUA SAM modified chip. (**C**) The overlay interaction plot of different concentration of immobilized antibodies (100 µg/ml, 40 µg/ml, 20 µg/ml) with Stxb (10 µg/ml).

The immobilization of anti-Stxb antibodies on the 11-MUA-modified sensor chip was performed using two distinct approaches: (1) oriented immobilization through protein G mediation and (2) non-oriented immobilization via direct covalent coupling. The immobilization process began with activation of the carboxyl groups on the 11-MUA SAM using a mixture of 400 mM EDC and 100 mM NHS. Following activation, anti-Stxb antibodies were introduced and covalently attached to the sensor surface through amine coupling chemistry. To minimize non-specific binding and block any remaining NHS-ester groups, the surface was subsequently treated with 1 M ethanolamine (pH 8.5). Figure 2A presents the 10-step immobilization profiles for both oriented and non-oriented methods, demonstrating the distinct kinetic patterns of each approach. For the non-oriented immobilization method, we systematically evaluated three different anti-Stxb concentrations (20, 40, and 100 µg/mL) to optimize surface antibody density. As shown in Fig. 2B, these experiments revealed concentration-dependent immobilization profiles. Comparative analysis of the binding signals (Fig. 2C) identified 40 µg/mL as the optimal antibody concentration, providing the strongest interaction signal while maintaining surface functionality. This optimized concentration was subsequently used for all comparative studies between oriented and non-oriented immobilization approaches.

### Direct detection Shiga toxin method

#### Interaction of Stxb antigen with the immobilized anti-Stxb on SPR

**Fig. 3:**
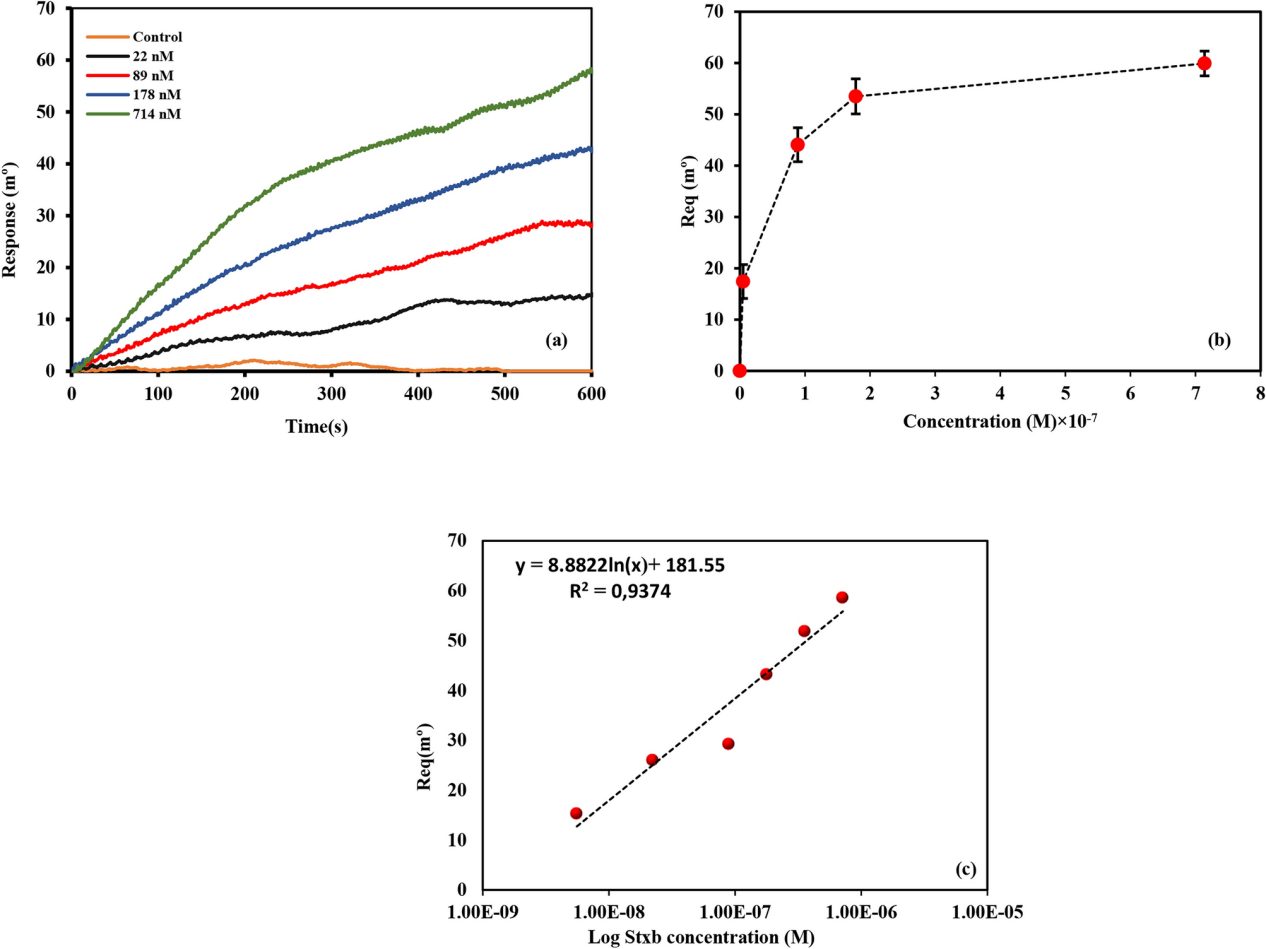
(**A**) The overlay interaction plot of different concentration of Stxb (22 nM– 714 nM) with 40 µg/mL immobilized antibody. (**B**) Langmuir isotherm plot of equilibrium angle (Req) versus Stxb concentration for 3 repetition of the interaction. (**C**) Logarithmic plot of equilibrium angle (Req) versus Stxb concentration.

The interaction between Stxb antigen and surface-immobilized anti-Stxb antibodies was investigated through systematic SPR analysis. A concentration series of Stxb antigen (22–714 nM) was introduced over the sensor surface functionalized with 40 μg/mL anti-Stxb. Figure 3A presents the real-time binding responses, demonstrating concentration-dependent signal enhancement with increasing antigen levels (Fig. S3 presents the full plot for the interaction). The binding characteristics were further analyzed through: (1) Langmuir isotherm analysis plotting equilibrium response (R_eq_) against Stxb concentration (Fig. 3B), with triplicate measurements showing reproducible binding behavior and (2) Logarithmic transformation of the dose–response relationship, which revealed a linear correlation between SPR signal and Stxb concentration (Fig. 3C). This quantitative analysis confirmed the specificity and concentration-dependent nature of the antibody-antigen interaction, validating the SPR platform for sensitive Shiga toxin detection. The linear dynamic range observed in the logarithmic plot suggests the method’s suitability for quantitative analysis across clinically relevant concentrations.

### Assay repeatability evaluation

**Fig. 4:**
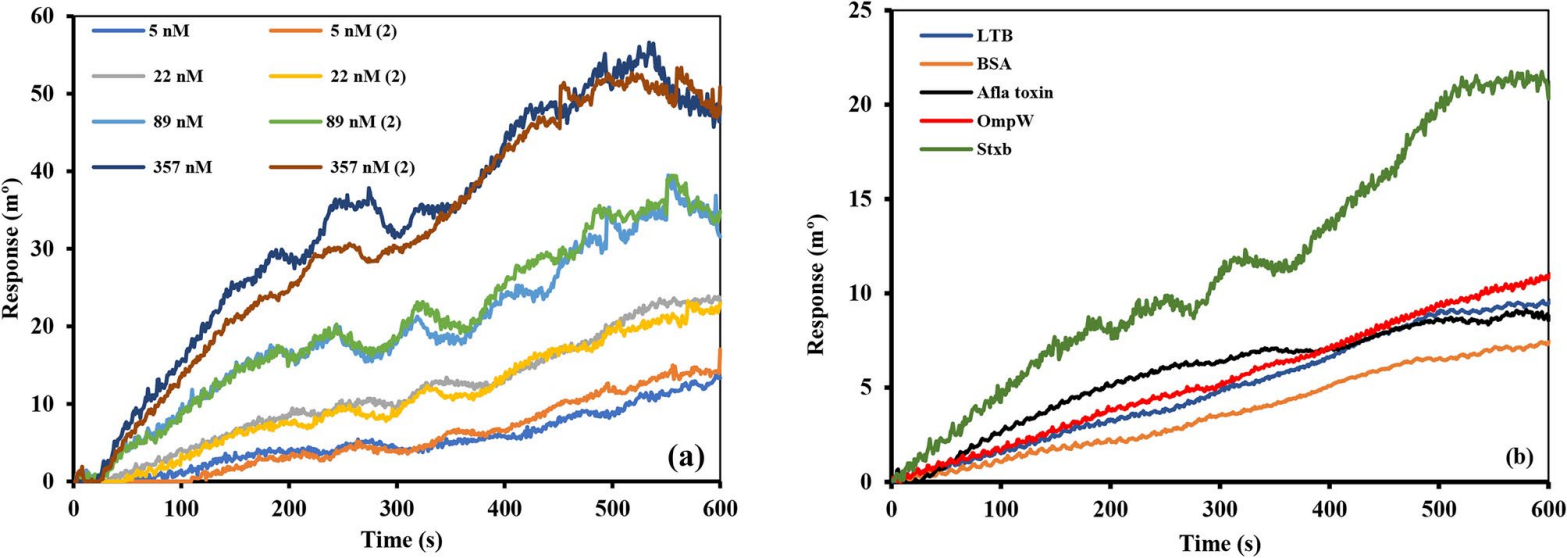
(**A**) Two intra-assay precision of the Stxb interaction (5 nM to 357 nM) with the 40 µg/mL immobilized antibody. (**B**) The comparative overlay plot of 22 nM of Stxb, Afla toxin, BSA, LTB and OmpW on immobilized anti-Stxb.

The repeatability of antibody-antigen interactions was evaluated through multiple experimental replicates conducted at different time points. This was achieved by repeatedly testing fixed antigen concentrations against immobilized antibodies. These repeated measurements of antigen binding to the sensor surface represent intra-assay precision (repeatability). Figure 4A presents the results of two independent intra-assay precision tests examining Stxb interactions across a concentration range of 5–357 nM with surface-immobilized antibodies (40 µg/mL). Complete sensorgrams are provided in Fig. S4.

### Evaluation of SPR sensor specificity

To assess the binding specificity of our SPR platform, we conducted comparative analyses using Stxb antigen and four control proteins (OmpW, LTB, AFLA toxin, and BSA) at equivalent concentrations (5 µg/mL or 22 nM). The sensor demonstrated minimal cross-reactivity with non-target proteins, showing response signals of only 7–10 mº for control proteins (BSA: 7 mº, AFLA toxin: 8 mº, LTB: 9 mº, OmpW: 10 mº). In marked contrast, the specific interaction with Stxb antigen generated a significantly stronger response (22 mº), representing a 2.2- to 3.1-fold increase over background signals (Fig. 4B; complete sensorgrams in Fig. S5).

### Interaction of Stxb antigen with the immobilized proG/anti-Stxb on SPR

**Fig. 5:**
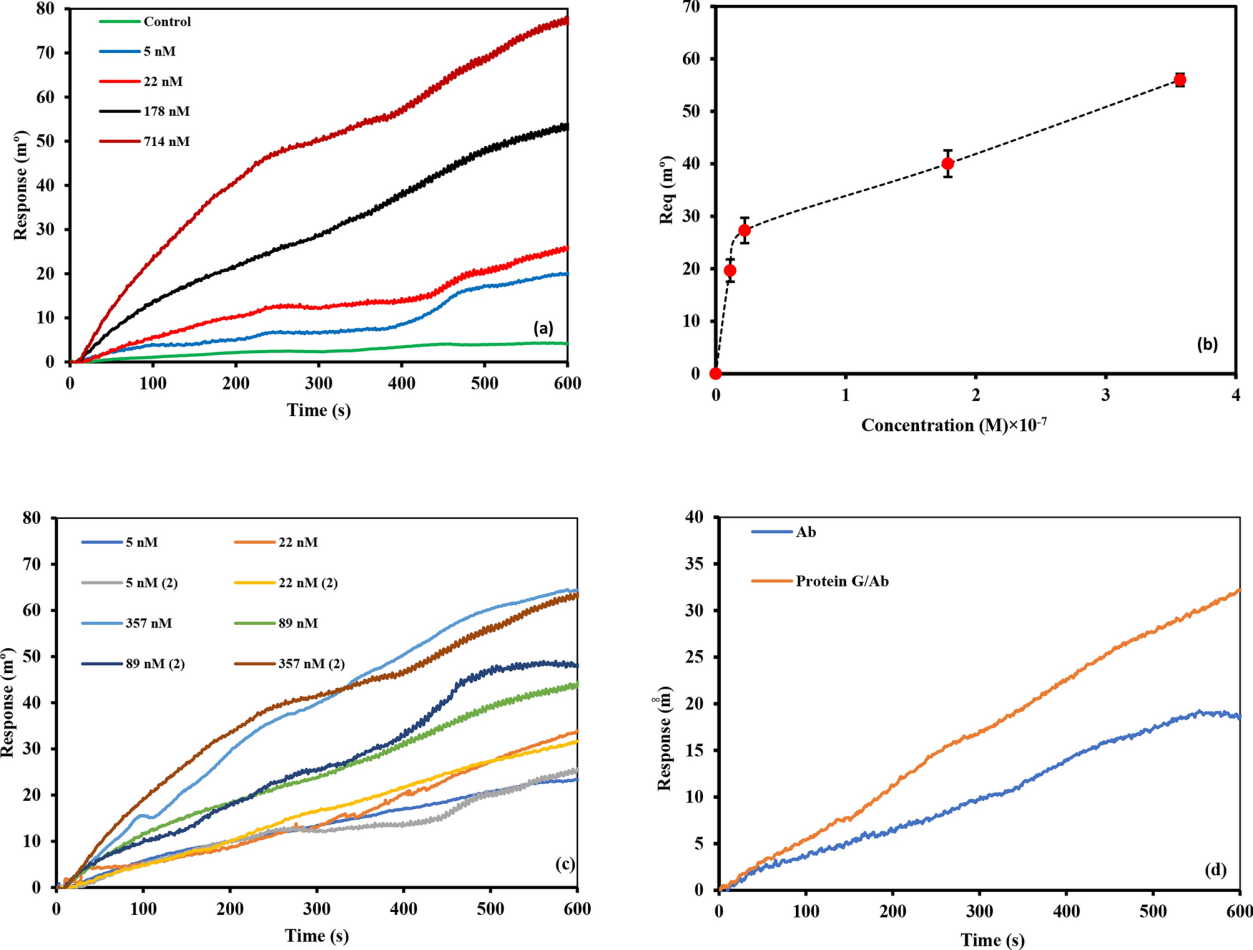
(**A**) The interaction of different concentrations of antigen samples (5 nM to 714 nM) with immobilized proG/anti-Stxb. (**B**) Langmuir isotherm plot of equilibrium angle (Req) versus Stxb concentration. (**C**) Two intra-assay precision of the Stxb interaction (5 nM to 357 nM) with the 40 µg/mL immobilized antibody. (**D**) The comparative interaction plot of Stxb (22 nM) with immobilized anti-Stxb with two methods of immobilization on 11MUA SAM and protein G-11MUA SAM.

The SPR sensor surface was functionalized with a protein G-11-MUA self-assembled monolayer (SAM), enabling oriented immobilization of anti-Stxb antibodies through specific Fc region binding. This configuration optimally exposed the antigen-binding Fab domains toward the solution phase, minimizing steric hindrance and maximizing binding site accessibility. Comprehensive binding analysis was performed across a concentration gradient of Stxb antigen (5–714 nM). Figure 5A presents the real-time binding responses (complete sensorgram overlays in Fig. S6), while Fig. 5B shows the corresponding Langmuir isotherm analysis plotting equilibrium response (R_eq_) against Stxb concentration with triplicate measurements. Figure 5C presents two intra-assay precision of the Stxb interaction (5—357 nM) with the 40 µg/mL immobilized antibody (complete sensorgram overlays in Fig. S7). As shown in Fig. 5D, the protein G-mediated immobilization approach demonstrated superior performance compared to conventional 11-MUA SAM attachment when detecting 22 nM Stxb, confirming the effectiveness of oriented antibody presentation for sensitive toxin detection.

### Interaction of anti-Stxb with the immobilized Stxb on SPR

**Fig. 6:**
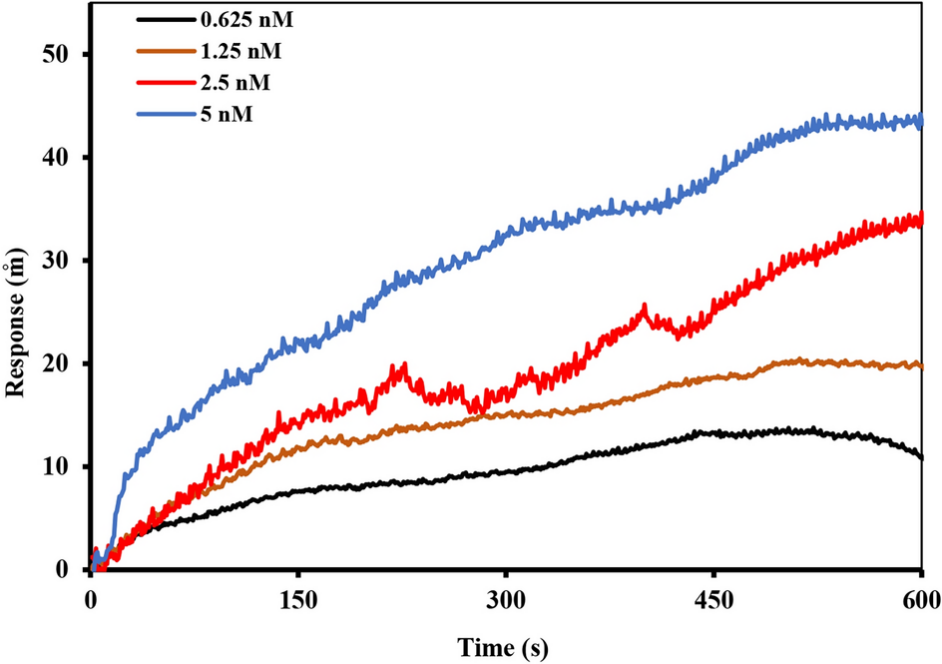
The interaction plot of different concentrations of anti-Stxb samples (0.625 nM to 5 nM) with immobilized 40 μg mL^−1^ Stxb.

The binding interactions between anti-Stxb antibodies and surface-immobilized Stxb antigen were characterized through SPR analysis (Fig. 6, with complete sensorgram overlays in Fig. S8). In this experimental configuration, 40 μg/mL of Stxb was immobilized on an 11-MUA-modified sensor chip following the established protocol. Various concentrations of anti-Stxb (0.625–5 nM) were then introduced to assess the binding characteristics. Kinetic analysis yielded a K_D_ of 10 nM, providing quantitative measurement of the interaction strength. This experimental design served two primary objectives: first, to evaluate the maximal antibody binding capacity to surface-immobilized antigen, and second, to assess potential effects of immobilization on antibody functionality by comparing K_D_ values obtained through different antibody immobilization approaches. The study provides important insights into how antibody immobilization strategies influence molecular recognition events, with particular focus on maintaining antigen-binding affinity. By employing an antigen-immobilized configuration as a comparative model, we were able to systematically evaluate the impact of surface attachment on antibody activity and binding performance.

## Discussion

In this study, antibodies were immobilized on the sensor surface for Shiga toxin detection. Among the tested antibody concentrations (20, 40, and 100 µg/mL), the strongest interaction signal was achieved with 40 µg/mL. This suggests that an optimal antibody density is critical for antigen–antibody interaction—excessive or insufficient antibody quantities can impair binding efficiency. A lower surface density of antibodies reduces steric hindrance, thereby enhancing the sensor’s sensitivity compared to densely packed, randomly oriented antibodies.

Also, covalent immobilization of antibodies on the surface compared with physical immobilization has the advantages of simply controlling the amount of immobilization and making the sensor reusable^24^. However, the direct immobilization of the antibody at the sensor surface leads to the possibility of blocking the active site of some immobilized antibodies. So, to reduce the possibility of hindering these points, we used protein G as a linker between the surface and antibody, and the affinity interaction of antigen and antibody and the sensitivity of the SPR sensor were estimated^11,25^. Also, in order to obtain the binding affinity of the antibody and antigen in the state where the antibody does not have a blocked point, the antigen was immobilized on the sensor surface, and its obtained K_D_ was compared with two previous antibody immobilization methods.

The interaction between the Stxb antigen and the immobilized anti-Stxb yielded a LOD of 2.03 nM (28 ng/mL). Also, according to the"kinetic evaluation software version 5.4,"K_D_ and B_max_ values as kinetic parameters were measured at 37 nM and 56 mº, respectively. Compared to ELISA as one of the most common methods for detecting Shiga toxin (LOD: 90 ng/mL with the same antibody-antigen^21^), our SPR-based assay demonstrated superior sensitivity. Furthermore, the SPR sensor offers significant advantages over ELISA and microbial culture methods, including faster detection times^24^.

In the alternative immobilization method used in this study – employing protein G for oriented anti-Stxb immobilization – the LOD for Stxb antigen interaction with immobilized ProG/anti-Stxb was estimated at approximately 9.8 ng/mL. This represents a 2.9-fold improvement compared to covalent immobilization. The measured K_D_ of 16 nM – notably lower than the covalent immobilization method’s 37 nM – demonstrates protein G’s effectiveness as a molecular linker for Stxb detection in SPR. This 57% reduction in K_D_ value directly reflects improved antigen–antibody binding affinity due to proper antibody orientation. The oriented immobilization showed a modest 7% increase in total binding capacity (B_max_: 60 vs 56 m°), yet achieved significantly stronger binding affinity (57% lower K_D_). This demonstrates that protein G’s key benefit isn’t merely increasing antibody density, but rather optimizing: antigen-binding site accessibility, antibody structural integrity and binding site availability per unit area. In similar research, Taheri et al. used two detection methods employing protein G for oriented antibody immobilization, which proved more sensitive than covalent immobilization^26^. Makaraviciute et al. demonstrated a 3.5-fold amplification in the hGH detection signal using protein G compared to random antibody orientation^27^. Also, Vashist et al. utilized protein A/G for CRP detection, revealing that protein G-mediated orientation maximized both antibody density on the sensor surface and binding-site accessibility^28^. Table 1 compares SPR with other Shiga toxin detection methods, highlighting their respective LODs.

**Table 1:** Some Shiga toxin detection methods and their LODs.

Method	LOD	Receptor	Sample	Reference
ELISA	90 ng/mL	Antibody	Synthetic sample	^21^
Antibody microarray	110 ng/mL	Antibody	Synthetic sample	^29^
Optical biosensor	6.23 μg/mL	Antibody	Beef lysate	^30^
LSPR	10 ng/mL	Gb_3_	Synthetic sample	^31^
SPRi	50 ng/mL	Antibody	Synthetic sample	^32^
SPR	28 ng/mL	Antibody	Synthetic sample	Current study
SPR	9.8 ng/mL	Antibody/ Protein G	Synthetic sample	Current study

**Fig. 7:**
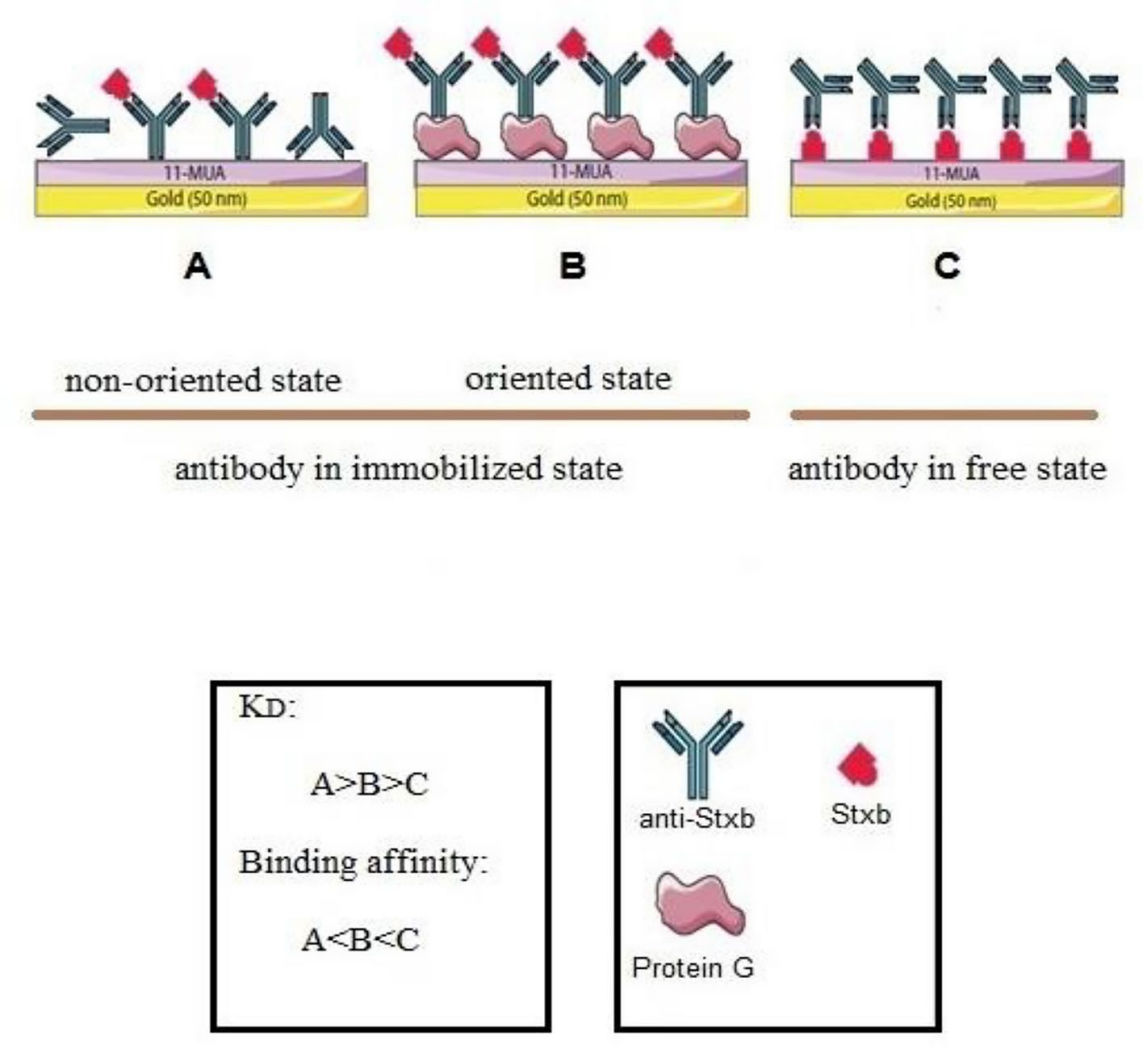
Schematic illustration of antibody in two different states: non-immobilized (free state) and immobilized (oriented and non-oriented immobilized state) on the sensor surface. The binding affinity between the antibody and antigen is maximized in the non-immobilized state. In the orientated immobilized state, the adverse impact of immobilization is diminished, resulting in increased antigen binding to the antibody.

To evaluate repeatability, we performed repeated measurements of fixed antigen concentrations on the sensor at different time points. The results demonstrated minimal variation in response for each concentration, confirming high reproducibility of the Shiga toxin detection method for both immobilization strategies Sensor. Specificity was validated by testing against the target Stxb antigen and control proteins/toxins at equivalent concentrations. The sensor exhibited excellent antibody-antigen binding specificity and minimal non-specific adsorption to the sensor surface. The > 2-fold signal differential between target and non-target proteins meets established criteria for specific detection in biosensor applications, validating the platform’s selectivity for Shiga toxin monitoring. Also, this study investigated how antibody immobilization on sensor surfaces affects antibody activity and antigen-binding affinity. The experimental approach involved immobilizing antigens on the surface and evaluating the dissociation constant (K_D_). The free-state antibody exhibited a K_D_ of 10 nM for its target antigen. Comparative analysis of K_D_ values revealed that immobilization reduced binding affinity, covalent immobilization caused a 3.7-fold decrease in affinity and oriented immobilization showed a 1.6-fold reduction. This affinity reduction likely results from steric hindrance and partial blocking of antibody binding sites during immobilization, which interferes with optimal antibody-antigen interactions. The results demonstrate that while effective binding can be achieved in both oriented and non-oriented configurations, the orientation and density of immobilized molecules significantly affect the measured interaction parameters. This comparative approach offers valuable information for optimizing biosensor surfaces to maintain biological activity while maximizing detection sensitivity. Figure 7 illustrates the antibody in both free and immobilized states (showing both oriented and non-oriented configurations on the sensor surface) and Table 2 summarizes the critical experimental results obtained in this investigation.

**Table 2:** Comparative analysis of antibody immobilization strategies: Binding parameters for Stxb-anti-Stxb interaction across different antibody states.

Type of antibody state	K_D_	B_max_	LOD
Ab in non-oriented immobilized state	37 nM	56 m º	28 ng/mL
Ab in oriented immobilized state	16 nM	60 m º	9.8 ng/mL
Ab in free state	10 nM	-	-

## Conclusions

This report presents the SPR assay as a rapid and sensitive method for the direct detection of Shiga toxin. The assay uses thiolated protein G as a foundation to create a layer of highly oriented antibodies. Additionally, the study compares two immobilization methods for Shiga toxin detection. We immobilized bare anti-Stxb and protein G/anti-Stxb on the sensor surface, followed by injection of Stxb antigens into the biosensor. The covalent immobilization of antibodies yielded a K_D_ of 37 nM and an LOD of 28 ng/mL*,* demonstrating good sensitivity and selectivity for Shiga toxin detection. In contrast, the protein G-mediated oriented immobilization of antibodies resulted in a K_D_ of 16 nM and an LOD of 9.8 ng/mL, indicating enhanced binding affinity and detection sensitivity*.* Higher binding affinity between the toxin and antibody correlates with an improved detection limit. The difference in detection limits between the two methods arises from the detrimental effect of direct immobilization, where non-oriented antibody attachment can block active sites, rendering them inaccessible to the antigen. To assess the impact of immobilization on antigen–antibody binding affinity, we compared the antibody’s free-state affinity (K_D_ = 10 nM) with both immobilized states. The results indicate that protein G, as a linker, yields a K_D_ value closer to the free-state antibody, confirming that it preserves a higher number of active antibodies compared to direct immobilization. Therefore, oriented immobilization of antibodies on the surface is recommended to enhance the sensor’s sensitivity and efficiency in detecting Shiga toxin. In this study, the potential of surface plasmon resonance (SPR) for Shiga toxin detection was demonstrated. Compared to conventional bacterial detection methods such as ELISA and other techniques, SPR offers advantages including higher speed, greater sensitivity, and a suitable detection limit.

## Data Availability

All data generated or analysed during this study are included in this published article.
